# A cluster randomized factorial trial of school-lunch salad bars and marketing on elementary students’ objectively measured fruit and vegetable consumption

**DOI:** 10.1186/s12966-025-01758-z

**Published:** 2025-05-26

**Authors:** Marc A. Adams, Michael Todd, Mindy L. McEntee, Tsung-Yen Yu, Punam Ohri-Vachaspati, Timothy J. Richards, Meg Bruening

**Affiliations:** 1 College of Health Solutions, Arizona State University, 425 N. 5 Th Street, Phoenix, AZ 85004 USA; 2 School of Technology for Public Health, Arizona State University, 425 N. 5 Th Street, Phoenix, AZ USA; 3 Edson College of Nursing and Health Innovation, Arizona State University, 550 N. 3Rd Street, AZ Phoenix, 85004 USA; 4 W. P. Carey Morrison School of Agribusiness, Arizona State University, 7231 E. Sonoran Arroyo Mall, Mesa, AZ 85212 USA; 5 Department of Nutritional Sciences, Chandlee Laboratory, College of Health and Human Development, The Pennsylvania State University, 110, University Park, University Park, PA 16802 USA

**Keywords:** Child nutrition, School lunch, Fruit, Vegetables, Cluster randomized trial, Intervention

## Abstract

**Background:**

Schools in the United States play a crucial role in promoting healthy eating habits. Despite numerous public health efforts, children’s consumption of fruits and vegetables (FVs) still fall short of recommended amounts. Advocates have promoted school lunch salad bars as an effective strategy to increase primary students’ FV consumption, but rigorous research has lagged behind their efforts. This study evaluated the effectiveness of introducing school lunch salad bars, FV marketing, and the combination of both on elementary students’ objectively measured fresh FV selection and consumption.

**Methods:**

A cluster-randomized factorial trial was conducted among 13 elementary schools from 12 public school districts participating in the U.S. National School Lunch Program with more than 50% of students eligible for free or reduced lunch. Schools were assigned randomly to one of four conditions: Salad Bar with FV Marketing (*n* = 4), Salad Bar-only (*n* = 3), FV Marketing-only (*n* = 3), and wait-listed control (*n* = 3). The conditions were assessed at three measurement waves. Students’ (*N* = 3,080) aggregated fresh FV selection and consumption were measured via digital scales (grams) using objective plate waste methodology. Zero-inflated negative binomial models were used to examine differences in consumption (accounting for excess zeros and overdispersion) by condition and wave.

**Results:**

No significant differences were observed for students selecting FVs across any condition. In contrast, after ten weeks of exposure, stand-alone school lunch salad bars significantly increased students’ consumption of FV (IRR = 1.84, 95% CI 1.12, 3.04) compared to the wait-listed control. Findings for FV marketing alone suggested increased consumption (IRR = 1.60, 95% CI 0.97, 2.64) relative to control, but were non-significant and inconclusive. Salad bars in combination with FV marketing showed the strongest effect on FV consumption relative to the wait-list control, with a significant increase observed at ten weeks (IRR = 2.07, 95% CI 1.29, 3.31).

**Conclusions:**

Stand-alone salad bars are effective at increasing elementary students’ FV consumption after a minimum of ten weeks. The combined intervention of salad bars and FV marketing demonstrated the greatest improvement in FV consumption. These findings support the promotion of salad bars in schools as a strategy to increase FV consumption among students.

**Trial registration:**

ClinicalTrials.gov Identifier: NCT03283033 (preregistered on: 9/14/2017).

**Supplementary Information:**

The online version contains supplementary material available at 10.1186/s12966-025-01758-z.

## Introduction

Despite numerouspublic health initiatives, children’s consumption of fruits and vegetables (FVs) in the United States still fall far short of recommended amounts [1–3]. Nationally, only 7.1% and 2% of children on average meet recommendations for fruit and vegetables, respectively [2]. Schools play a crucial role in promoting healthy eating habits and providing essential nutrients for optimal learning, growth, and development, as elementary children consume up to two meals and a snack at school daily. Before the COVID-19 pandemic, the National School Lunch Program (NSLP) served 28–29 million children per day, providing over 4.8 billion lunches in school cafeterias per year, with over 70% being free or reduced in price for low-income families at risk of malnutrition [4]. As of the fiscal year 2024, the NSLP continues to serve a similar number (29.6 million) of students lunch per day [4].

The Healthy, Hunger Free Kids Act (HHFKA) of 2010 introduced important changes to the NSLP, requiring schools to offer a greater variety and quantity of FVs to children [5]. To promote FVs, schools use a range of strategies, including salad bars, school gardens, posters and other marketing materials, and FV tastings. Many school nutrition staff view salad bars as an effective means of enhancing FV variety and consumption [6], even after COVID-19 [7].

Unlike pre-portioned FV items typically offered or served on the main service line by food service staff, self-service salad bars are standalone units—located either inside or outside of the service line—often equipped with cold wells and restocked daily, where students may self-serve fruits, vegetables, or other meal components [8]. To qualify as part of a reimbursable meal, selected items must meet grade-appropriate minimum portion size requirements [8]. Salad bars are referred to by various terms in school settings, including produce bars, fresh fruit and vegetable bars, condiment bars, among others [6].

According to the Centers for Disease Control and Prevention (CDC) and independent evaluations, the use of self-serve salad bars in elementary schools nationally grew from 17% in 2006 to 29% in 2014 [9, 10]. More recent data suggest the proportion of schools is much higher [11], but these data are limited. The increased use of salad bars is likely due to efforts by the USDA, CDC, and professional associations who advocate for salad bars to increase FV access in compliance with the HHFKA [12]. For example, Salad Bars to School (SB2S) has installed over 6100 salad bars in schools as of 2024 with the aim of having one in every school [13]. In 2015, the U.S. House of Representatives proposed “Salad Bars in Schools Expansion Act” aimed to provide federal funding for establishing school salad bars nationwide, but it was never passed [14]. A new bill (HR 7344) was reintroduced in the U.S. Congress in 2024 “to promote the use of salad bars in schools participating in the school lunch program” and maintains that salad bars are an effective strategy to increase FV consumption in elementary, middle, and high schools [15].

A narrative review found many critical gaps in the evidence for the efficacy of school lunch salad bars for increasing FV consumption [16]. To date, most of the research is limited to elementary schools, with a handful of studies conducted in middle and high schools [16–20]. The literature includes several observational studies, but only two quasi-experimental pre-post evaluations of salad bar interventions in elementary schools. Slusser et al. found that the introduction of a multicomponent intervention that included a salad bar in elementary schools significantly increased the reported 24-h frequency of daily fruit and vegetable consumption among children from low-income households. [21] Bean et al. found salad bars increased the selection, but unexpectedly decreased the consumption of FVs among children in two Title I elementary schools. [22] These quasi-experimental studies—lacking proper control conditions—offer limited causal evidence to support the use of salad bars to increase FV consumption. While some observational studies found modest positive differences between schools with versus without salad bars on FV selection and consumption, these differences may be due to unmeasured factors such as differences in the schools or students who have access to salad bars [16, 23].

Although theories support the marketing of FVs, the combined effect of FV marketing and salad bar implementation on actual intake has not been systematically examined. The impact of implementing new salad bars, with or without accompanying marketing efforts, in cafeterias on FV consumption remains unknown [24, 25]. To address these gaps and strengthen the evidence base, research is need to assess the impact of introducing salad bars on consumption and waste in schools where salad bars do not yet exist, both with and without nutrition marketing interventions. Such studies require rigorous methods, such as using objective measures and including proper comparison groups, to minimize effects of potential bias, secular trends, and alternative explanations for observed results to develop more robust evidence. Given the widespread support and proliferation of salad bars in schools, studies that address these areas could provide school decision-makers with critical information needed to make evidence-based health and financial decisions while serving millions of students through school lunch programs.

To date, there has been no rigorous study on the effects of introducing salad bars on student FV consumption, the impact of FV marketing materials on the use of salad bars, the combined influence of salad bars and FV marketing, or the replicability of effects across different grade levels. Given the far-reaching implications of any changes to the school meals program, it is crucial to have clear and robust evidence to inform policy decisions. This cluster-randomized controlled trial aimed to address several previous limitations by evaluating the efficacy of offering school lunch salad bars, freely-available marketing materials promoting FVs, and the combination of both to increase elementary students’ selection and consumption of fresh FV items [26]. We hypothesized that students in schools assigned to receive salad bars would increase their FV selection and intake, independent of their assignment to the marketing intervention. Additionally, we hypothesized that students in schools assigned to receive FV marketing would increase their FV selection and intake, irrespective of whether they were assigned to receive a salad bar. Finally, we hypothesized that the combined intervention of salad bars and marketing would yield the greatest improvements in selection and consumption compared to the wait-listed salad bar with wait-listed marketing conditions.

## Methods

### Study design and sample

**Fig. 1:**
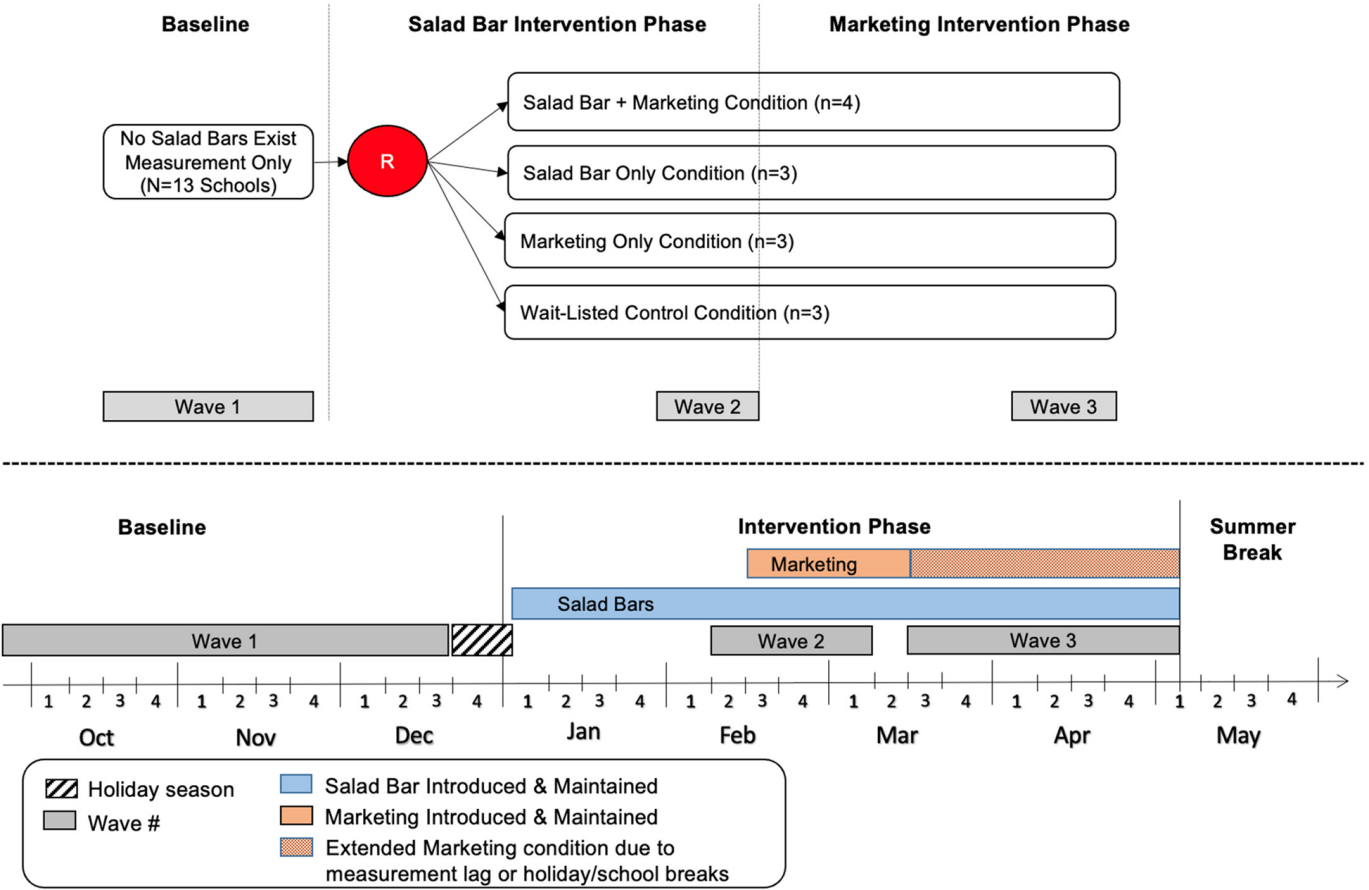
Overview of study design, intervention conditions, and timeline of data collection activities in elementary schools

The study methods and measurement process have been published previously [26]. Briefly, this cluster-randomized factorial controlled trial sampled 13 Arizona elementary schools from 12 public school districts participating in the National School Lunch Program. Schools that participated in the National School Lunch Program with 40% or more of students eligible for free or reduced lunch and not currently using a salad bar were eligible for the study. None of the participating schools had an existing salad bar in the six years before the study, and schools with an open campus for lunch or enrolling fewer than 120 students were excluded from participating. A block randomized (block size = 4) factorial design was employed to assign eligible schools to one of four intervention conditions: Salad Bar with FV Marketing (i.e., both intervention components; *n* = 4), Salad Bar-only (*n* = 3), FV Marketing-only (*n* = 3), and a wait-listed measurement-only control (*n* = 3). Schools allocated to the Salad Bar-only, FV Marketing-only, and measurement-only control conditions and wait-listed for intervention components (i.e., marketing and/or salad bar) were provided these components at the end of the study. The imbalance in the number of schools randomized to conditions resulted from recruiting a thirteenth school to guard against school-level attrition; however, all schools ultimately remained in the study. Fidelity assessments were conducted approximately every two weeks at both intervention and wait-list control schools to ensure to adherence to the protocol and to monitor for alternative interventions. Figure 1 shows the design, intervention components, and measurement timeline.

The study was conducted in elementary schools during the 2017–2018 (*n* = 5 schools) and 2018–2019 (*n* = 8) academic years. A single baseline measure (Wave 1) of students’ FV selection and consumption was conducted in each school between August and December during the academic school year, depending on the school and study staff’s availability. Schools were randomly assigned to study conditions after completing the baseline measure. Additional measurement waves occurred later in the same academic year. Wave 2 occurred for all intervention and wait-listed control schools approximately 6–9 weeks after the onset of the salad bar intervention for all schools, and Wave 3 occurred approximately 10–17 weeks after the introduction of marketing materials (see lower panel of Fig. 1). Researchers instructed school staff to maintain their usual lunch procedures and not to alter their usual protocol during the research periods.

School principals acted as surrogates for parents (in loco parentis) and provided informed consent before data collection. Students were randomly selected via a computerized process from a sampling frame of all enrolled students provided by each school. Random selection was conducted prior to school visits and stratified proportionally by grade level. The students were blinded to the purpose of the overall study at all waves and informed that the researchers were merely interested in students’ lunch habits. Project staff were blinded at baseline (before random allocation) to a school’s intervention condition, but due to the nature of the study, they could not be blinded to conditions post random allocation at Waves 2 or 3. Each student provided verbal assent before participating in the study. The study was preregistered at ClinicalTrials.gov: NCT03283033 (date of registration: 9/14/2017). Arizona State University’s Institutional Review Board approved the study protocols (STUDY00005882).

#### Salad bar intervention

**Fig. 2:**
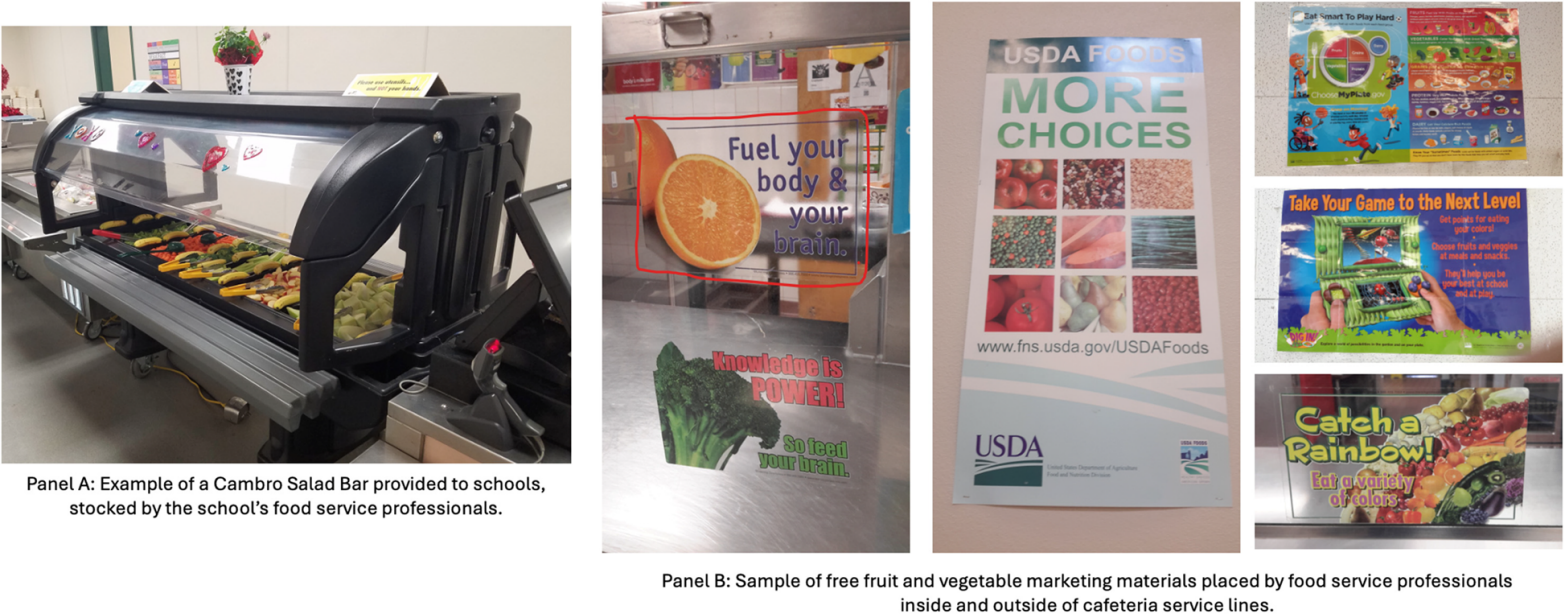
Examples of salad bar and fruit and vegetable marketing materials provided to the intervention conditions

One Cambro Versa freestanding, mobile-insulated salad bar was provided to each intervention school assigned to the salad bar condition for use beginning after winter break (see Fig. 2). In preliminary work, we identified this type of salad bar as the most commonly used in schools that offer salad bars [17]. Because schools varied in size and cafeteria design, schools chose where to locate their salad bar in the cafeteria (i.e., inside the service line before the point of purchase or in the eating area after the point of purchase). School food service workers also choose how to stock and use the salad bar throughout the study. Research staff provided school lunch staff with brief training on salad bar sanitation, menu and layout (but not marketing), preparation and food handling, recordkeeping, use for meeting reimbursable meal requirements, and student food handling etiquette. The salad bars were in place for a minimum of 6 weeks until Wave 2 occurred and remained in use through the end of the school year in the 7 schools assigned to the Salad Bar with Marketing and Salad Bar-only conditions. Schools assigned to these conditions kept the salad bars following the conclusion of the study.

#### Marketing intervention

Prior to implementing the marketing intervention component, we pretested freely available marketing materials from 30 campaigns promoting fruit and vegetables, including: USDA’s Food and Nutrition Service [27], Team Nutrition [28], Team FNV [29], the National Dairy Council [30], and the American Heart Association [31], among others. Campaigns included posters and table tents varying in size, along with spoken announcements. We surveyed a national convenience sample of school nutrition professionals (*n* = 1546) and asked them to rank them on perceived attractiveness/appeal, comprehension, relevance, motivation/persuasion, and uniqueness of marketing materials. School nutrition professionals’ top-ranked materials were further pretested with a sample of local youth (*n*= 61; 51% female, 52.5% Hispanic; 98% participated in free/reduced-price lunch) living in the Phoenix, Arizona metro area [32]. Details of this preliminary work have been reported elsewhere [32]. Based on the results of this pretesting, school nutrition managers participating in the current RCT were offered a menu of free, pre-existing marketing materials that had been rated highly by both nutrition professionals and youth in Arizona. Schools randomized to the marketing condition received these materials after completing Wave 2. The delayed onset of the marketing intervention allowed for the evaluation of both the sequential and combined effects of the salad bar and marketing components. With the support of research staff, school nutrition professionals placed the marketing materials in the cafeteria for a minimum of 4 weeks or until Wave 3 occurred. Figure 2 provides marketing material examples. A total of 7 schools were randomized to receive FV marketing materials and 6 were randomized to the wait-list marketing condition. Schools kept the FV marketing materials to use after the study concluded.

### Objective measures of FV selection and consumption

Objective “plate waste” measures were collected over three measurement waves (at a pre-randomization baseline, 6 weeks post salad bar implementation, and 4 weeks post marketing intervention). Randomly selected students were identified and assented while queuing in the hot lunch line, and researchers provided assented students with a pre-weighted barcoded tray that was otherwise identical to the trays used at the school. The colored barcode was linked to the student ID through the study application on a tablet during the assent process. Students were asked to eat lunch normally and not to share food or discard any food or trash before they finished their meals. Students at each school proceeded through the lunch line according to the school’s typical lunchroom procedures. After exiting the lunch line and before eating, students with barcoded trays were redirected to a weigh station.

Trained staff operated each pre-lunch weigh station and measured FV selection by obtaining two weights (grams) with calibrated Cardinal Detecto PZ30W scales accurate to two grams: 1) the entire tray (including beverage, entrée, fresh fruits, fresh vegetables, grain, and dessert) and 2) fresh FV items separately from other lunch items (i.e., warm or hot FVs as part of the entrée were not included). Several approaches were used to isolate fresh FV items from the entrée, depending on the entrée and serving approach used for fresh FV items (e.g., pre-portioned in non-salad bar schools). In all cases, the fresh FV items were weighed in isolation. Additionally, project staff also independently photographed the entire tray and the fresh FV items. FV weights for each tray were recorded in the aggregate and documented by the photography of scales and trays.

After students finished eating their meal, staff intercepted students with barcoded trays before they discarded their trays in trash receptacles. Students were thanked and allowed to select an age-appropriate incentive for participating in the study and completing the measurement process (e.g., a small toy, sunglasses, earbuds). Trays were set aside for post-lunch weighing and photography outside of view, following the procedure described above. At post-lunch, any remaining fresh FV items were scraped onto scales for individual post-lunch weighing. Post-lunch fresh FV weights included fruit rinds (e.g., peels from whole fruits) if present. To ensure accuracy, photographs of weights for pre-lunch and post-lunch were entered in triplicate by trained RAs and weights in grams recoded for statistical analysis. Discrepancies were adjudicated by a fourth senior staff member. Student FV selection was operationalized as the aggregate amount (grams) of fresh FV taken from the service line and/or salad bar. FV consumption was operationalized by subtracting the student’s gram weight at post-lunch for FV items only from the gram weight of FVs selected at pre-lunch. For each measure, a net weight was determined by factoring out serving trays, cups, bags, measurement cups, and paper plates from each measurement. Student’s use of condiments or salad dressing at either pre- or post-lunch was measured by a dichotomous indicator (present/absent). We measured all students with the same standardized measurement approach, regardless of study condition or grade level. Therefore, any potential reactivity due to measurement activities or staff were not expected to differ across conditions.

### Sociodemographic information

Before the day of baseline data collection, schools and/or districts provided the study team with sociodemographic information for the school population consisting of each student’s identification number, sex, race/ethnicity, grade level, and free/reduced price lunch eligibility.

### Analytic approach

*Power analyses*. Formulas for tests of between-condition differences in change from baseline to any given post-intervention wave for serial samples [33] indicated that a complete-case sample size of 12 schools (6 schools per Salad Bar condition or 3 schools per Salad Bar x Marketing combination) and *N* = 50 student contacts per school at each of 3 waves would afford power of 0.92 to detect a mean difference as small as 25 g, given standard deviation of 50 g (i.e., Cohen’s *d* = 0.50), assuming alpha of 0.05 and an autocorrelation between temporally adjacent school means of 0.80. In anticipation of up to 20% loss of students during measurement activities (e.g., a student’s tray is not intercepted before disposing it), we planned for 63 student contacts (50/(1–0.20) = 63) per school at each wave (Nc x Np × 3 waves = 2,268 total student contacts per grade level) to ensure a sufficient complete-case sample.

Prior to estimation of models examining intervention effects, the distribution of each outcome measure was examined using plots and univariate descriptive statistics, and preliminary generalized linear mixed models (GLMMs) were evaluated to determine which error distribution and link function were most appropriate to the data. Model comparison was conducted in the performance package [34] in R version 4.3.0 [35]. For FV selection, the distribution of values (in grams) showed strong positive skewness, such that a model with a negative binomial error distribution (with log link) yielded superior model performance relative to a standard linear model with normally distributed errors (and an identity link). For FV consumption, the distribution of values (in grams) showed a preponderance of zeros (18.2% of observations) and strong positive skewness. In model comparisons, a zero-inflated negative binomial model (ZINB; with log link) best fit the data. ZINB models comprise two sub-models, a conditional model for the non-zero consumption values (i.e., grams of FV consumed, given any consumption) and a zero-inflation (ZI) model for zero vs. non-zero consumption values (i.e., probability of no consumption vs. any consumption).

Preliminary examination of data also revealed that students at one school (School 17) in the Marketing-only condition during Wave 2 data collection showed extreme zero values for FV attributable to the school offering of a 3-bean salad at lunch, resulting in an anomalous pattern of values for both FV selection and consumption. We arrived at this conclusion based on comparisons of the observed values from the affected school’s second wave as well as to those for all other school-waves in the sample. Accordingly, we estimated GLMMs for this outcome (see detailed description below) using three different approaches: (a) analysis of observed data values for all schools and measurement waves; (b) analysis of a dataset with all of School 17’s Wave 1 values (*n* = 86) carried forward, replacing the observed Wave 2 values (*n* = 70); and (c) analysis of 100 datasets, each containing 70 observations randomly sampled (with replacement) from School 17’s Wave 1 values, which were carried forward to replace the school’s Wave 2 observed values.

Following an intent-to-treat approach, all schools randomized to conditions were retained in the final analyses. To test intervention effects on two outcomes (i.e., FV selection and consumption), we estimated GLMMs with school-level fixed effect terms for the study arm (wait-listed control [Control], Salad Bar-only, Marketing-only, Salad bars with Marketing [SB + Marketing]), measurement wave (Waves 1, 2, and 3), and their interaction. Fixed effects of child-level background covariates (sex, race/ethnicity, grade in school, free or reduced-price lunch) were also included along with a dichotomous indicator coding for the presence (vs. absence) of salad dressing in pre-lunch images of the trays. To account for clustering at the school and school-wave levels, random school-level and school-within-wave intercept terms were also included. Consistent with best practices [36], to characterize the degree of clustering (non-independence) of outcome values at the school and school-wave levels, we estimated intraclass correlation coefficients (ICCs) using methods for count outcomes [37] as implemented in the iccCounts package [38]. For FV selection, the observed school-level ICC was 0.108, and the school-wave-level ICC was 0.231. For FV consumption, the observed school-level ICC was 0.067, and the school-wave-level ICC was 0.157. GLMMs were estimated using the glmmTMB package [39]. Each of the 100 datasets generated via imputation (carrying forward) random samples of School 17’s Wave 1 values to Wave 2, were analyzed separately using GLMMs, and estimates from these 100 were pooled and tested using the mice package [40]. To test the significance of the overall Condition x Wave interaction, the fit of the final (full) model was compared to that of a model with only main (linear) effects via a Wald F-test using the D1 function in the mice package. Estimation of the final models was followed by estimation of model-predicted means and probabilities and contrasts to probe differences in patterns of change within and across study conditions using the emmeans package [41].

## Results

### Sample demographics

**Fig. 3:**
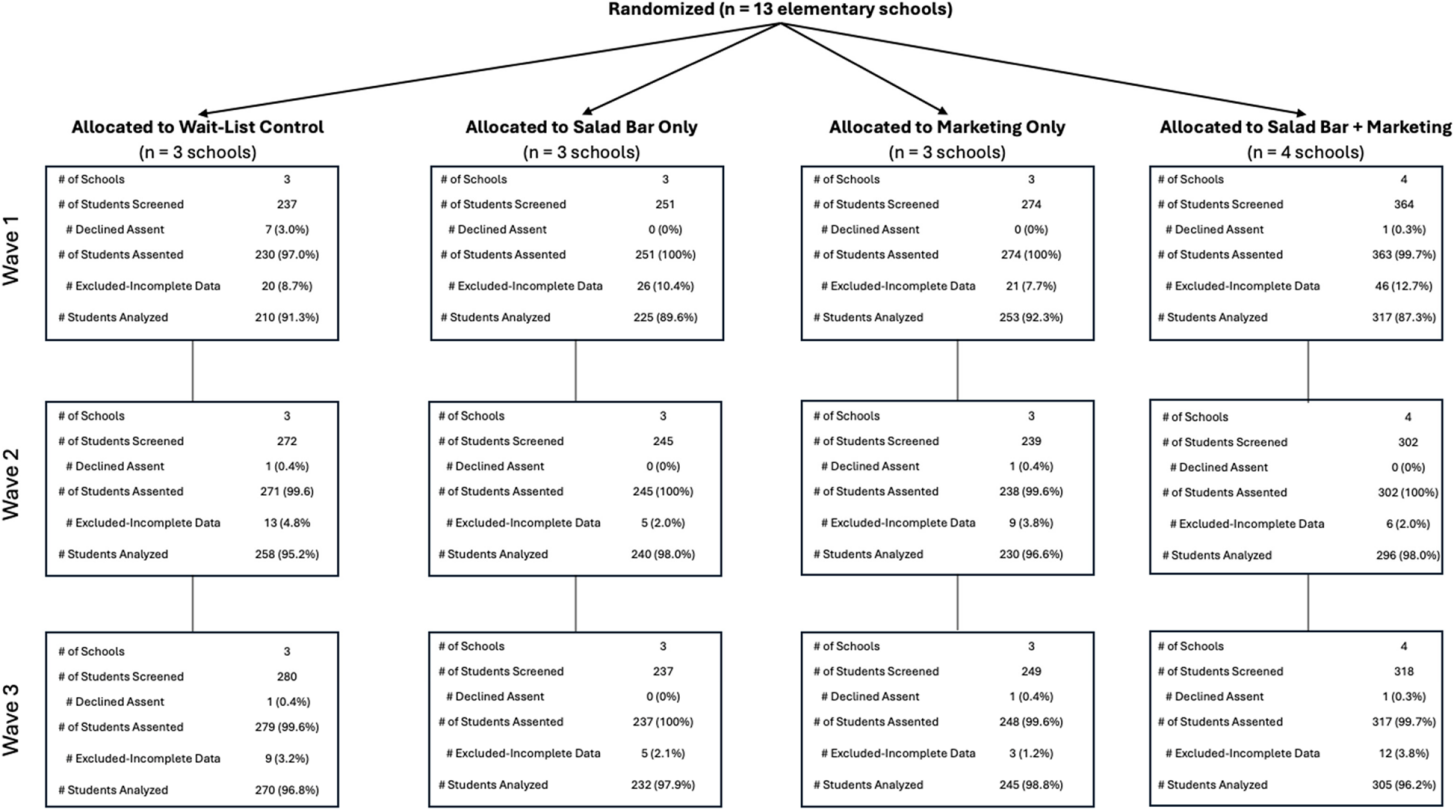
CONSORT diagram

Figure 3 presents the number of student observations (*N* = 3,080) by each study arm across three measurement waves, and Table 1 shows demographic characteristics of the sample. Supplementary Table A compares the pooled demographic characteristics of participating schools’ overall student populations to those of the analytic sample. Compared to the broader student populations of these 13 schools, our sample was overrepresented by students eligible for free or reduced-price lunches, third graders, and Hispanic White or Hispanic students of unknown race. Among the total analyzed sample (Table 1), 48.5% were female (*n* = 1,494) and the distribution across grades was fairly even, with the largest proportions in the 3rd grade (22.3%, *n* = 686) and the smallest in the 1 st grade (16.3%, *n* = 502). The largest race/ethnicity subgroup was Hispanic White, comprising 41.6% of the sample (*n* = 1,280), followed by Non-Hispanic White at 25.6% (*n* = 787). Hispanic students of unknown race accounted for 14.2% (*n* = 438), while other racial/ethnic categories such as Hispanic non-white/more than one race, American Indian/Alaska Native, Asian or Pacific Islander, and Black/African American represented smaller portions of the sample at 5.8%, 3.2%, 2.7%, and 7.0%, respectively. Notably, 79.8% of the students were eligible for free or reduced-price lunch (*n* = 2,458), indicating a significant proportion of the sample came from schools serving students from lower socioeconomic backgrounds.

**Table 1:** Observed data: sample demographic characteristics by condition and wave (*n* = 3080 observations from 13 schools)^a^

Variable	Wave 1				Wave 2				Wave 3				
	Control *n (%)*	SB *n (%)*	Mktg *n (%)*	SB + Mktg*n (%)*	Control *n (%)*	SB *n (%)*	Mktg*n (%)*	SB + Mktg *n (%)*	Control *n (%)*	SB *n (%)*	Mktg*n (%)*	SB + Mktg *n (%)*	Total*n (%)*
Female	101 (48.1)	97 (43.1)	131 (51.8)	141 (44.5)	137 (53.1)	108 (45.0)	128 (55.9)	127 (42.9)	142 (53.0)	99 (42.7)	132 (53.9)	151 (49.5)	1494 (48.5)
Grade													
1 st	35 (16.7)	20 (8.9)	39 (15.4)	65 (20.5)	51 (19.8)	24 (10.0)	25 (10.9)	60 (20.3)	57 (21.1)	31 (13.4)	44 (18.0)	51 (16.7)	502 (16.3)
2nd	25 (11.9)	26 (11.6)	59 (23.3)	58 (18.3)	47 (18.2)	28 (11.7)	54 (23.6)	61 (20.6)	56 (20.7)	36 (15.5)	52 (21.2)	58 (19.0)	560 (18.2)
3rd	46 (21.9)	58 (25.8)	58 (22.9)	71 (22.4)	49 (19.0)	56 (23.3)	59 (25.8)	71 (24.0)	54 (20.0)	55 (23.7)	48 (19.6)	61 (20.0)	686 (22.3)
4 th	50 (23.8)	65 (28.9)	49 (19.4)	56 (17.7)	54 (20.9)	67 (27.9)	52 (22.7)	53 (17.9)	56 (20.7)	61 (26.3)	52 (21.2)	63 (20.7)	678 (22.0)
5 th	54 (25.7)	56 (24.9)	48 (19.0)	67 (21.1)	57 (22.0)	65 (27.1)	39 (17.0)	51 (17.2)	47 (17.4)	49 (21.1)	49 (20.0)	72 (23.6)	654 (21.2)
Race/ethnicity													
Non-Hispanic White	8 (3.8)	42 (18.7)	83 (32.8)	120 (37.9)	13 (5.0)	51 (21.2)	76 (33.2)	115 (38.9)	21 (7.8)	51 (22.0)	84 (34.3)	123 (40.3)	787 (25.6)
Hispanic, White	116 (55.2)	97 (43.1)	104 (41.1)	124 (39.1)	142 (55.0)	89 (37.0)	88 (38.4)	91 (30.7)	150 (55.6)	86 (37.1)	102 (41.6)	91 (29.8)	1280 (41.6)
Hispanic, Unknown race	35 (16.7)	57 (25.3)	6 (2.4)	22 (6.9)	40 (15.5)	72 (30.0)	15 (6.6)	37 (12.5)	41 (15.2)	64 (27.8)	16 (6.5)	33 (10.8)	438 (14.2)
Hispanic, non-white/> 1 race	12 (5.7)	13 (5.8)	21 (8.3)	15 (4.7)	15 (5.8)	9 (3.8)	20 (8.7)	16 (5.4)	15 (5.6)	12 (5.2)	17 (6.9)	14 (4.6)	179 (5.8)
American Indian/Alaska Native	14 (6.7)	6 (2.7)	1 (0.4)	7 (2.2)	25 (9.7)	3 (1.2)	2 (0.9)	10 (3.4)	19 (7.0)	1 (0.4)	3 (1.2)	7 (2.3)	98 (3.2)
Asian or Pacific Islander	5 (2.4)	3 (1.3)	2 (0.8)	17 (5.4)	5 (1.9)	3 (1.2)	3 (1.3)	9 (3.0)	8 (3.0)	7 (3.0)	5 (2.0)	15 (4.9)	82 (2.7)
Black/African American	20 (9.5)	7 (3.1)	36 (14.2)	12 (3.8)	18 (7.0)	13 (5.4)	25 (10.9)	18 (6.1)	16 (5.9)	11 (4.7)	18 (7.3)	22 (7.2)	216 (7.0)
Free/Reduced-price lunch	200 (95.2)	194 (86.2)	167 (66.0)	231 (72.9)	245 (95.0)	209 (87.1)	152 (66.4)	231 (78.0)	249 (92.2)	190 (81.9)	160 (65.3)	230 (75.4)	2458 (79.8)

*SB* Salad Bar-only condition, *Mktg* Marketing-only condition, *SB* + *Mktg* Salad Bar and Marketing condition

^a^Compared to the overall student populations of the 37 participating schools, our sample was overrepresented by students eligible for free or reduced-price lunches, third graders, and Hispanic White or Hispanic students of unspecified race (see Appendix Table A)

#### Fruit and vegetable selection

Tables 2 and 3 show observed and model-estimated values for both any selection and the amounts selected, by condition and wave, respectively. None of the conditions differed significantly in any (vs. none) or total amount of FV selected at baseline, and the values for FV selected remained generally stable across waves within study arms. In the SB arm, however, there was an unexpected, though not significant, decrease from Wave 1 (134.3 g) to Wave 2 (109.7 g), and a return to the baseline value (134.4 g) at Wave 3. Focused between-group comparisons of change in amount of FV selected revealed no significant differences (see Table 4).

**Table 2:** Observed proportions, means, and standard deviations for FV selection and consumption measures by condition and wave (*n* = 3,080 observations from 13 schools)

Study condition	Wave 1	Wave 2	Wave 3
Any FV selected^a^			
Wait-listed Control	0.96 (0.19)	0.95 (0.21)	0.87 (0.34)
Salad bar-only	0.99 (0.11)	0.99 (0.11)	0.97 (0.16)
Marketing-only	1.00 (0.00)	0.76 (0.43)	0.96 (0.20)
Salad bar + Marketing	0.99 (0.10)	0.88 (0.32)	0.92 (0.27)
FV selected (g)^b^			
Wait-listed Control	129.4 (82.6)	132.4 (67.0)	128.1 (69.3)
Salad bar-only	128.1 (37.8)	107.4 (53.7)	135.0 (61.9)
Marketing-only	101.0 (62.5)	87.0 (60.8)	113.7 (38.8)
Salad bar + Marketing	111.4 (62.2)	117.8 (69.5)	123.8 (76.6)
Any FV consumption^a^			
Wait-listed Control	0.90 (0.31)	0.81 (0.39)	0.67 (0.47)
Salad bar-only	0.87 (0.34)	0.85 (0.35)	0.85 (0.35)
Marketing-only	0.89 (0.32)	0.59 (0.49)	0.89 (0.31)
Salad bar + Marketing	0.88 (0.32)	0.80 (0.40)	0.82 (0.39)
FV consumed, including zero values (g)^b^			
Wait-listed Control	65.0 (55.4)	43.6 (45.4)	32.4 (43.7)
Salad bar-only	45.9 (45.8)	50.1 (40.9)	57.8 (54.6)
Marketing-only	36.2 (48.6)	20.1 (29.9)	41.1 (36.7)
Salad bar + Marketing	41.4 (47.6)	51.8 (51.2)	55.3 (58.6)
FV consumed, excluding zero values (g)^b^			
Wait-listed Control	72.6 (53.7)	53.5 (44.7)	48.6 (45.5)
Salad bar-only	53.0 (45.2)	58.7 (38.2)	67.7 (53.1)
Marketing-only	40.8 (49.8)	34.3 (32.2)	46.0 (35.8)
Salad bar + Marketing	46.9 (48.1)	64.4 (49.4)	67.7 (58.0)

^a^Proportions and standard deviations

^b^Means and standard deviations

**Table 3:** Model-estimated means for FV selection (pre-lunch FV weights) by condition and wave, with contrast estimates of within-condition change in mean FV selection (*n* = 3,080 observations from 13 schools)

Study condition	Mean Amount of FV selected (g)			Within-condition contrast across waves		
	Wave 1	Wave 2	Wave 3	Wave 2 vs. Wave 1	Wave 3 vs. Wave 1	Wave 2 vs. Wave 3
	*M (95% CI)*	*M (95% CI)*	*M (95% CI)*	*IRR (95% CI)*	*IRR (95% CI)*	*IRR (95% CI)*
Wait-listed Control	128.5 (86.4, 191.2)	137.9 (92.8, 204.8)	133.0 (89.5, 197.5)	1.07 (0.71, 1.61) *p* = 0.735	1.04 (0.69, 1.55) *p* = 0.869	0.96 (0.64, 1.45) *p* = 0.861
Salad Bar-only	134.3 (90.4, 199.7)	109.7 (73.8, 163.2)	134.4 (90.4, 199.8)	0.82 (0.54, 1.23) *p* = 0.329	1.00 (0.67, 1.50) *p* = 0.998	1.22 (0.82, 1.84) *p* = 0.328
Marketing-only	97.8 (65.9, 145.2)	116.0 (78.0, 172.6)	119.6 (80.5, 177.7)	1.19 (0.79, 1.78) *p* = 0.413	1.22 (0.81, 1.84) *p* = 0.332	1.03 (0.68, 1.55) *p* = 0.884
Salad Bar + Marketing	112.1 (79.5, 158.0)	109.0 (77.2, 153.7)	118.7 (84.2, 167.3)	0.97 (0.68, 1.38) *p* = 0.875	1.06 (0.74, 1.51) *p* = 0.751	1.09 (0.76, 1.55) *p* = 0.636

Estimates are adjusted for student’s gender, race/ethnicity, grade, free or reduced (vs. full price) lunch status, and whether student’s lunch selection included salad dressing

*M *Model-estimated mean, *95% CI* 95% confidence interval, *IRR* Incidence rate ratio (ratio of model-estimated means), *p p*-value for test of significance of *IRR*

**Table 4:** Tests of pairwise condition x time interaction contrasts reflecting between-condition differences in change in FV selection (pre-lunch FV weights) across waves

Target condition	Reference condition		
	Wait-listed Control	Salad Bar-only	Marketing-only
	*IRR (95% CI)*	*IRR (95% CI)*	*IRR (95% CI)*
**Wave 2 vs. Wave 1**			
Salad Bar-only	0.76 (0.43, 1.35) *p* = 0.353		
Marketing-only	1.10 (0.62, 1.97) *p* = 0.733	1.45 (0.82, 2.58) *p* = 0.205	
Salad Bar + Marketing	0.91 (0.53, 1.55) *p* = 0.720	1.19 (0.70, 2.04) *p* = 0.526	0.82 (0.48, 1.41) *p* = 0.470
**Wave 3 vs. Wave 1**			
Salad Bar-only	0.97 (0.54, 1.72) *p* = 0.909		
Marketing-only	1.18 (0.66, 2.10) *p* = 0.570	1.22 (0.69, 2.17) *p* = 0.494	
Salad Bar + Marketing	1.02 (0.60, 1.75) *p* = 0.933	1.06 (0.62, 1.81) *p* = 0.837	0.87 (0.51, 1.48) *p* = 0.600
**Wave 3 vs. Wave 2**			
Salad Bar-only	1.27 (0.72, 2.26) *p* = 0.414		
Marketing-only	1.07 (0.60, 1.90) *p* = 0.820	0.84 (0.47, 1.50) *p* = 0.558	
Salad Bar + Marketing	1.13 (0.66, 1.93) *p* = 0.657	0.89 (0.52, 1.52) *p* = 0.669	1.06 (0.62, 1.81) *p* = 0.842

All contrasts are calculated as (Target condition minus Reference condition) x (Later wave minus Earlier wave) and are adjusted for student’s gender, race/ethnicity, grade, free or reduced (vs. full price) lunch status, and whether a student’s lunch selection included salad dressing. Wald F(6, 3050.0) = 0.34, *p* =.918

IRR values are incidence rate ratios reflecting between-condition comparisons (ratios) of between-wave comparisons (ratios) of model-estimated mean FV consumption values and associated 95% confidence intervals

*IRR* Incidence rate ratio, *95% CI* 95% confidence interval, *p p*-value for test of significance of *IRR*

#### Fruit and vegetable consumption

Table 2 presents observed values for both any consumption and grams of consumption (with and without zero values) by condition and Wave. Model-estimated means for FV consumption (given any consumption) and probabilities of any FV consumption are summarized in Tables 5 and 6. From Wave 1 to Wave 3, Table 5 Part A shows consumption decreased in the Control condition by 32% (IRR = 0.68, 95% CI = 0.47, 0.97) and increased in the intervention conditions, with the greatest increase (40% from Wave 1 to Wave 3) seen in the SB + Marketing condition (IRR = 1.40; 95% CI = 1.03, 1.90). Consumption increased more modestly in the SB-only (25%) and Marketing-only (9%) conditions. Table 5 Part B shows the probability of any consumption decreased appreciably across waves in the Control condition (by 17% from Wave 1 to Wave 3, OR = 0.20; 95% CI = 0.07, 0.52, *p* = 0.001) but was generally steady across waves in the intervention conditions.

**Table 5:** Model-estimated mean FV Consumption (given any consumption) and predicted probabilities of any FV consumption by condition and wave, with contrast estimates for within-condition change in amount of FV consumed and probability of any FV consumption (*n* = 3,080 observations from 13 schools)

**Part A. Amounts of FV Consumed by Elementary Students by Condition and Wave**						
	Estimated mean amount of FV consumed (g)			Within-condition contrast across waves		
	Wave 1	Wave 2	Wave 3	Wave 2 vs. Wave 1	Wave 3 vs. Wave 1	Wave 3 vs. Wave 2
Study condition	*M (95% CI)*	*M (95% CI)*	*M (95% CI)*	*IRR (95% CI)*	*IRR (95% CI)*	*IRR (95% CI)*
Wait-Listed Control	67.4 (45.1, 100.8)	50.5 (33.9, 75.3)	45.6 (30.5, 68.3)	0.75 (0.53, 1.07) *p* = 0.108	0.68 (0.47, 0.97) *p* = 0.032	0.90 (0.63, 1.29) *p* = 0.570
Salad Bar-only	48.8 (32.7, 72.8)	53.1 (35.6, 79.3)	60.8 (40.8, 90.6)	1.09 (0.77, 1.55) *p* = 0634	1.25 (0.88, 1.77) *p* = 0.220	1.14 (0.80, 1.63) *p* = 0.454
Marketing-only	36.5 (24.6, 54.2)	41.1 (27.4, 61.8)	39.6 (26.6, 59.0)	1.13 (0.79, 1.60) *p* = 0.515	1.09 (0.77, 1.54) *p* = 0.644	0.96 (0.67, 1.38) *p* = 0.839
SB + Marketing	40.1 (28.3, 56.7)	49.2 (34.6, 69.9)	56.0 (39.6, 79.4)	1.23 (0.90, 1.70) *p* = 0.193	1.40 (1.03, 1.90) *p* = 0.031	1.14 (0.84, 1.56) *p* = 0.409
**Part B. Probability of Any FV Consumption by Elementary Students by Condition and Wave**						
	Estimated probability of any FV consumption			Within-condition contrast across waves		
	Wave 1	Wave 2	Wave 3	Wave 2 vs. Wave 1	Wave 3 vs. Wave 1	Wave 3 vs. Wave 2
Study condition	*Pr (95% CI)*	*Pr (95% CI)*	*Pr (95% CI)*	*OR (95% CI)*	*OR (95% CI)*	*OR (95% CI)*
Wait-Listed Control	0.94 (0.88, 0.97)	0.89 (0.78, 0.94)	0.77 (0.63, 0.87)	0.46 (0.17, 1.26) *p* = 0.131	0.20 (0.07, 0.52) *p* = 0.001	0.42 (0.17, 1.05) *p* = 0.064
Salad Bar- only	0.92 (0.85, 0.96)	0.92 (0.86, 0.96)	0.92 (0.85, 0.96)	0.98 (0.37, 2.63) *p* = 0.971	1.02 (0.37, 2.76) *p* = 0.975	1.04 (0.39, 2.77) *p* = 0.946
Marketing-only	0.93 (0.85, 0.96)	0.86 (0.74, 0.93)	0.95 (0.89, 0.98)	0.51 (0.18, 1.43) *p* = 0.200	1.42 (0.48, 4.13) *p* = 0.525	2.80 (0.97, 8.05) *p* = 0.057
Salad Bar + Marketing	0.93 (0.88, 0.97)	0.88 (0.78, 0.93)	0.88 (0.80, 0.93)	0.53 (0.22, 1.27) *p* = 0.154	0.53 (0.22, 1.27) *p* = 0.156	1.01 (0.44, 2.30) *p* = 0.986

Estimates are adjusted for student’s gender, race/ethnicity, grade, free or reduced (vs. full price) lunch status, and whether student’s lunch selection included salad dressing

*M* Model-estimated mean, *95% CI* 95% confidence interval, *IRR* Incidence rate ratio (ratio of model-estimated means), *Pr* Model-estimated probability, *OR* Odds ratio (for comparison of model-estimated probabilities), *p p*-value for test of significance of *IRR* or *OR*

**Table 6:** Tests of pairwise condition x wave interaction contrasts reflecting between-condition differences in change over time in amount of FV consumed and probability of any FV consumption

Target condition	FV Amount Consumed(g)^a^			Any FV Consumption (vs none)^b^		
	Reference condition			Reference condition		
	Wait-Listed Control	Salad Bar-only	Marketing-only	Wait-Listed Control	Salad Bar-only	Marketing-only
	**Wave 2 vs. Wave 1**					
	*IRR (95% CI)*	*IRR (95% CI)*	*IRR (95% CI)*	*OR (95% CI)*	*OR (95% CI)*	*OR (95% CI)*
Salad Bar-only	1.45 (0.88, 2.39) *p* = 0.141	-	-	2.12 (0.52, 8.66) *p* = 0.294	-	-
Marketing-only	1.50 (0.91, 2.45) *p* = 0.112	1.04 (0.62, 1.71) *p* = 0.894	-	1.10 (0.26, 4.64) *p* = 0.902	0.52 (0.12, 2.17) *p* = 0.366	-
Salad Bar + Marketing	1.64 (1.03, 2.61) *p* = 0.039	1.13 (0.70, 1.80) *p* = 0.618	1.09 (0.68, 1.75) *p* = 0.725	1.14 (0.30, 4.32) *p* = 0.843	0.54 (0.14, 2.02)* p* = 0.358	1.04 (0.27, 4.06) *p* = 0.950
	**Wave 3 vs. Wave 1**					
	*IRR (95% CI)*	*IRR (95% CI)*	*IRR (95% CI)*	*OR (95% CI)*	*OR (95% CI)*	*OR (95% CI)*
Salad Bar-only	1.84 (1.12, 3.04) *p* = 0.017	-	-	5.19 (1.28, 20.98) *p* = 0.021	-	-
Marketing-only	1.60 (0.97, 2.64) *p* = 0.063	0.87 (0.53, 1.43) *p* = 0.586	-	7.23 (1.70, 30.78) *p* = 0.007	1.39 (0.32, 6.03) *p* = 0.657	-
Salad Bar + Marketing	2.07 (1.29, 3.31) *p* = 0.002	1.12 (0.70, 1.79) *p* = 0.627	1.29 (0.81, 2.05) *p* = 0.285	2.72 (0.74, 10.03) *p* = 0.133	0.52 (0.14, 1.97) *p* = 0.340	0.38 (0.10, 1.50) *p* = 0.165
	**Wave 3 vs. Wave 2**					
	*IRR (95% CI)*	*IRR (95% CI)*	*IRR (95% CI)*	*OR (95% CI)*	*OR (95% CI)*	*OR (95% CI)*
Salad Bar-only	1.27 (0.77, 2.09) *p* = 0.352	-	-	2.44 (0.64, 9.34) *p* = 0.191	-	-
Marketing-only	1.07 (0.64, 1.77) *p* = 0.801	0.84 (0.51, 1.40) *p* = 0.506	-	6.60 (1.63, 26.65) *p* = 0.008	2.70 (0.63, 11.752) *p* = 0.179	-
Salad Bar + Marketing	1.26 (0.79, 2.02) *p* = 0.331	1.00 (0.62, 1.59) *p* = 0.988	1.18 (0.73, 1.91) *p* = 0.490	2.38 (0.70, 8.12) *p* = 0.166	0.97 (0.27, 3.52) *p* = 0.967	0.36 (0.10, 1.38) *p* = 0.135

All contrasts are calculated as (Target condition minus Reference condition) x (Later wave minus Earlier wave) and are adjusted for student’s gender, race/ethnicity, grade, free or reduced (vs. full price) lunch status, and whether a student’s lunch selection included salad dressing. Wald F(6, 3022.1) = 1.77, *p* =.100

*IRR* Incidence rate ratio, *OR* Odds ratio, *95% CI* 95% confidence interval, *p p*-value for test of significance of *IRR* or *OR*

^a^Values are incidence rate ratios reflecting between-condition comparisons (ratios) of between-wave comparisons (ratios) of model-estimated mean FV consumption values and associated 95% confidence intervals

^b^Values are odds ratios reflecting between-condition comparisons (ratios) of odd ratios for between-wave comparisons of model-estimated probabilities of any FV consumption and associated 95% confidence intervals

**Fig. 4:**
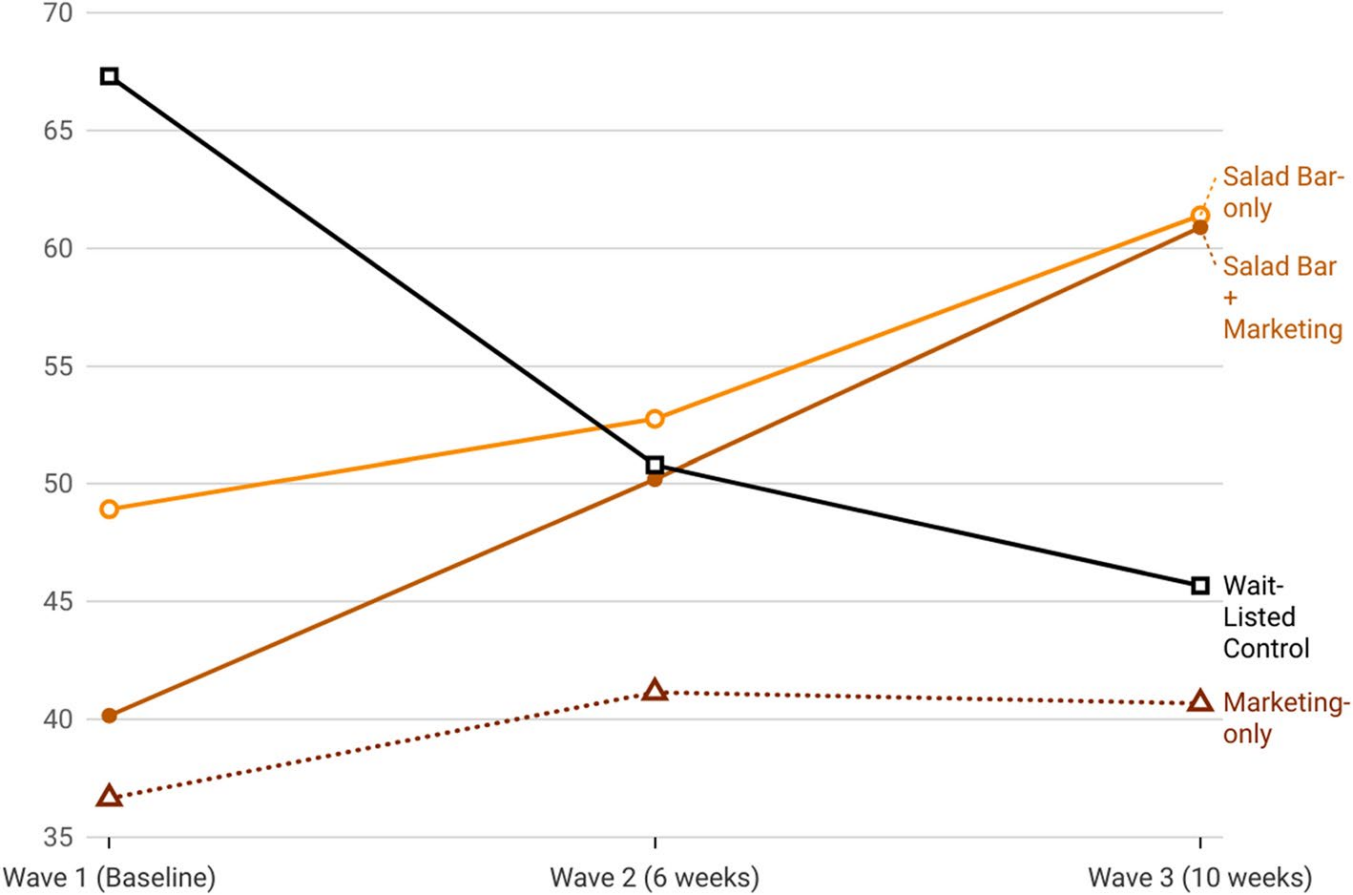
Model-estimated mean fruit and vegetable consumption (grams) by condition and wave

Table 6 and Fig. 4 show that changes in *amount of FV consumed* differed significantly across conditions. Between Wave 1 and Wave 2, the increase in FV consumption for the SB + Marketing condition was 64% greater than in the Control condition (Table 6: IRR = 1.64; 95% CI = 1.03, 2.61; *p* = 0.039). Table 5 Part A reveals the SB + Marketing condition showed a 23% increase in FV consumption (IRR = 1.23; 95% CI = 0.90, 1.70) from Wave 1 to 2, whereas the Control condition experienced a 25% decrease (IRR = 0.75; 95% CI = 0.53, 1.07). Table 6 shows, from Wave 1 to Wave 3, the SB + Marketing condition differed from the Control, IRR = 2.07; 95% CI = 1.29, 3.31; *p* = 0.002, likely resulting from a 40% increase SB + Marketing and 32% decrease in Control consumption (Table 5 Part A). The difference between the Salad Bar-only condition versus Control condition was also significant (IRR = 1.84; 95% CI = 1.12, 3.04; *p* = 0.017) from Wave 1 to Wave 3, with Salad Bar-only increasing by 25% over time. The difference between Wave 1 to Wave 3 change in the Marketing condition (9% increase) compared to Control, however, was not significant (IRR = 1.60, 95% CI = 0.97, 2.64; *p* = 0.063). No significant between-condition differences in the degree of change in FV consumption from Wave 2 to Wave 3 were observed.

Change in the probability of any FV consumption from Wave 1 to Wave 3 in the Salad Bar-only and Marketing-only conditions differed significantly from that in the Control condition (OR = 7.23; 95% CI = 1.70, 30.78, *p* = 007; see Table 6), reflecting the difference between the large decrease in the Control condition and the relative lack of change in the Marketing-only condition (see Table 5 Part B). The degree of change in consumption for the Marketing-only condition did not differ significantly from that for Control. Also, the change in probability of any FV consumption from Wave 2 to Wave 3 in the Marketing-only condition differed from that in the Control condition (OR = 6.60; 95% CI = 1.63, 26.65, *p* = 008). No between-condition differences in Wave 1 to Wave 2 change in probability of any FV consumption were observed.

No adverse effects were reported in any of the conditions.

## Discussion

Proponents of school lunch salad bars promote their use to increase elementary students’ fruit and vegetable consumption, but without rigorous evidence of their effectiveness. This cluster-randomized trial tested the effects of schools adopting salad bars with and without staggered FV marketing in the cafeteria to increase students’ selection and consumption of fruits and vegetables. The results of this trial partially support our hypotheses that students in schools assigned to receive salad bars or FV marketing materials increased their FV selection and intake. Contrary to our hypothesis, we found no evidence that adopting salad bars, FV marketing, or the combination of both increased students’ *selection* of FV. Regarding students’ consumption of FV, we found that salad bars after 10 weeks of exposure significantly increased the amount of FV students consumed relative to the wait-list control. The marketing-only condition did not increase FV consumption at any time point relative to the wait-list control. However, the combination of salad bars with FV marketing increased consumption, and this effect became stronger by the end of the intervention relative to the wait-list control condition.

Salad bars have been promoted by government agencies and advocates of school meals as a mechanism to increase students’ exposure to a wider variety of FV and to allow them to *find and choose* greater amounts of their preferred FV, resulting in less waste. Previous observational and quasi-experimental research suggests that FV consumption was partially due to increased selections, but the research was limited to observational studies. Students attending two elementary schools with salad bars selected more FV than students in two schools offering pre-portioned servings without salad bars (112 + −70 g vs 104 + −86g) [42]. This study occurred in 2005 before the HHFKA, which increased the variety and amounts offered to students, but it used plate waste measures similar to the current trial. In a single-group quasi-experimental study, salad bars increased FV selection (estimated by direct observation of digital photos of FV on trays) among children in two Title I elementary schools [22].

Contrary to previous studies, the current RCT did not support the hypothesis that introducing salad bars (either with or without FV marketing) significantly increased FV selection compared to a wait-list control at any of the three measurement waves. Baseline average amounts of FV selected by students ranged from 101 to 129 g. Although the amounts of FV selected by students varied across the waves and conditions of this study, none of the between condition differences were significant. Our control schools provided a strong counterfactual for evaluating the treatments on FV selection, and repeated measures after the onset of the intervention consistently showed no effect at each time point, supporting a robust inference that these interventions do not causally influence FV selection. It should be noted that food service workers decided how to use the salad bars (e.g., by moving existing items onto them or by adding new choices).

The observed variation in FV selection may be explained in part by the variability in schools and students within each condition. Food service managers selected foods to stock on the salad bar, which likely contributed to variability in the types of FV offered at both baseline and during the intervention period. This pragmatic approach was intentional, as it allowed the study to reflect real-world conditions. Food service managers are most familiar with their student populations, budget constraints, and changes in local food prices. While all schools participated in the NSLP and had greater than 50% of students eligible for free-and-reduced-price lunch, previous studies have observed that school and student characteristics influence FV selection, which may explain the non-significant variation [43]. The current prospective results suggest that salad bars, and FV marketing, are not influencing students’ selection of aggregate amounts of fruits or vegetables during lunch, but it is still possible that salad bars and marketing help to shift student preferences to select different types of or their favorite fruits or vegetables, without affecting the total amount selected.

The results reported here support the use of stand-alone salad bars for increasing fruit and vegetable *consumption* relative to a wait-list control. This effect was the positive direction in the first 6 weeks of exposure (i.e., through Wave 2) but was non-significant (Salad Bar-only, IRR = 1.45, *p* = 0.14). The change in FV consumption in the Salad Bar-only condition relative to Control only emerged as significant after students had a minimum exposure of 10 weeks (i.e., through Wave 3, IRR = 1.84, *p* = 0.017). Likewise, the effect on students’ consumption of any FVs (versus none) was positive, but not significant, through Wave 2, and only emerged as significant at Wave 3 (OR = 5.19, *p* = 0.021). These are important findings and suggest that the initial novelty of salad bars is not a concern. Rather the full impact of introducing salad bars takes at least 10 weeks to emerge for students in elementary schools. The behavioral mechanisms for this finding are unclear but may be a result of children requiring more instances of exposure or schools learning how to best use salad bars for increasing consumption. This research showing salad bars increase consumption is supported by previous observational research and quasi-experimental studies. For example, Slusser et al.’s multicomponent intervention with a salad bar in elementary schools found students from low-income households significantly increased their reported frequency of daily fruit and vegetable consumption [21] and a recent natural experiment found salad bars were associated with about a third of a cup more consumption of fruits at 4–6 weeks relative to non-exposed students [26]. However, the current study does not support the mixed findings in Bean et al.’s single-group intervention introducing salad bars to children attending two Title I elementary schools, which found the salad bars decreased FV consumption (estimated by direct observation of digital photos of FV on trays) over a one-month period. [22] Results from the current confirmatory trial demonstrate that a minimum implementation period of 10 weeks is necessary for salad bars alone to have measurable impacts on elementary’s students’ FV consumption.

Our hypotheses that FV marketing would increase FV consumption was partially supported. Notably, the onset of the marketing intervention component was intentionally delayed until after Wave 2. As a result, the effects of marketing alone or in combination with salad bars can only be assessed from Wave 3. Increasing the number of FV marketing items in schools and cafeterias in the absence of salad bars appears to have increased consumption, though not significantly, between baseline and Wave 3 (10 weeks) relative to control (IRR = 1.60, *p* = 0.063). Additionally, students significantly increased consumption of any (versus none) FVs from baseline to 10 weeks (OR = 7.23, *p* = 0.007) and from 6 to 10 weeks (OR = 6.60, *p* = 0.008). As expected, no significant increase was observed for marketing only at Wave 2 because the marketing intervention had not started.

The pattern of results reflects the implementation of the FV marketing conditions, as the onset of FV marketing alone started after Wave 2 measures and occurred for only 4 weeks. This design decision allowed the investigative team to disentangle any effects of salad bars and FV marketing and understand the sequence of modifying the cafeteria environment (i.e., adding salad bars before marketing) in the joint condition. While the effect of FV marketing on increasing *any *FV consumption is remarkable, its impact on the overall amount consumed was minimal. This suggests that while FV marketing may encourage students to try FVs, marketing alone does not substantially increase their overall FV intake. Given that marketing is often most impactful initially due to the novelty of messages and images, a four-week intervention (with posters, table tents, and school announcements) may have been insufficient in the elementary school context. Nonetheless, the results strongly suggest an intervention effect, even if not statistically significant, in terms of grams consumed. Notably, in our pretesting of the existing marketing materials for school nutrition professionals, both youth and nutrition professionals indicated a need for higher quality and more impactful messages for healthy eating consumption [32]. School nutrition staff in the current study selected marketing materials from a menu of options from the highest-ranked materials. It is possible that the dose and placement of messages could have been enhanced to increase the joint effect of salad bars and marketing. For example, since it appears that sufficient FVs are being offered and taken, more nuanced prompting of FV consumption after selection may be needed (e.g. table tents prompting consumption (not selection) at the lunch table). Additional research is needed to explore how to maximize marketing of nutrient-rich foods like FVs in cafeterias.

Regarding the joint contributions of salad bars and FV marketing materials, the study supported the hypothesis that this combination of components would increase FV consumption. The use of salad bars combined with FV marketing increased grams of FV consumed at both 6 weeks (IRR = 1.64, *p* = 0.039) and 10 weeks (IRR = 2.07, *p* = 0.002), demonstrating a stronger effect of added marketing exposure. As mentioned above, the FV marketing component of this condition did not start until after the Wave 2 measure (i.e., minimum 6 weeks), so the initial effects observed at the Wave 2 measure reflect the impact of the salad bars alone. These results confirm that this multi-component intervention – sequentially implementing salad bars followed by FV marketing – had a synergistic effect on improving FV consumption and should be recommended as an effective school-based strategy.

The NSLP affects millions of children daily. School lunch salad bars have become increasingly prevalent in the US schools as a mechanism for increasing FV consumption, with little prior evidence to support their use. For example, Salad Bars to School (SB2S) has supported over 6100 schools in obtaining salad bars, with the goal of having a salad bar in every single school. Congress authored a bill, “Salad Bars in Schools Expansion Act” (H.R. 2627) in 2015 and again in 2024, calling for federal funding to expand use and technical assistance for school salad bars nationally [14, 15]. The evidence from this study supports efforts by advocates and government officials to promote salad bars or salad bars alone with FV marketing as national strategy to help children increase their FV consumption during school lunch. Salad bars alone, as well as in combination with FV marketing, increased consumption by an average of 38 to 45 g (unadjusted) compared to the measurement-only control, under conditions requiring students to select a fruit or vegetable.

Methodological considerations: This study offers several methodological strengths including a diverse sample (80% eligible for free/reduced lunch) of students from 13 schools and 12 public school districts, a rigorous factorial approach to the intervention components, a wait-list comparison condition to account for potential secular trends and external confounding, an objective plate waste measure appropriate for evaluating consumption among children less than 11 years old, a 10 week intervention period with two standardized measurements during the intervention period, digital photographs of scale weights to ensure accuracy, and triplicate data entry of scale weights to minimize errors. Only one school participated in the Fresh Fruit and Vegetable Program, minimizing any concerns about a direct co-intervention possibility occurring in the schools and influencing our results.

Potential weaknesses should also be noted. Compared to the overall student populations of the 13 participating schools, our sample was overrepresented by students eligible for free or reduced-price lunches, third graders, and Hispanic White or Hispanic students of unspecified race. This overrepresentation may be attributable to our school and student recruitment strategy, which involved selecting schools with higher rates of free/reduced price lunch participation, and randomly sampling students prior to visiting the schools and assenting students waiting in the hot lunch line; students receiving free or reduced-price lunches are more likely to select the hot lunch option compared to students paying full price. Because we relied on schools for students’ demographic information, we encountered variation in the approach to race and ethnicity classifications. Specifically, several schools (not all of them) used a single self-report measure of race and ethnicity, with response options that included “Hispanic” in the options that included racial categories. Thus, when a parent selected “Hispanic” or “White,” the schools survey precluded the possibility for other categories, and this affected about 14% of our sample. We utilized existing FV marketing materials judged to be the most appealing to both a sample of school meal professionals and students. However, these existing static materials were dated in many instances (e.g., basketball stars from two decades ago), and not reflective of modern marketing students experience daily on social media or outside of the cafeteria. Food service managers, rather than study investigators, stocked the salad bars with fresh FV item, which could have introduced variability in desirability of FV items served across schools. This would be true for all study groups. We intentionally chose this approach because food service managers are most familiar with their students’ preferences, school’s budget, and local food prices. Given the geographic and demographic diversity of the urban and rural schools included in this study, we did not presume to know the most appropriate or appealing options for each setting. Fruits and vegetables included as part of the entrée were excluded, likely underestimating the total amount of FV consumed at lunch. When salad bars are present, the results reflect total amounts of FVs taken/consumed, including those offered from both the salad bar and service line. However, in our observations, the majority of FVs were on the salad bar when it was available. Future studies may examine how to better align FVs on the salad bar with students’ preferences in a given region. While this study was conducted in a single Southwest region of the US, the sample of schools served a demographically diverse population of lower-income minority students.

## Conclusion

Stand-alone school lunch salad bars are effective at increasing elementary students’ consumption of fruits and vegetables after a minimum of ten weeks of exposure. Increasing only FV marketing in the cafeteria was suggestive but not conclusive for increasing consumption. In contrast, implementing salad bars combined with adding FV marketing six weeks afterward showed the strongest effect on students’ consumption by study end. This study offers the most rigorous and definitive evidence to date on the effectiveness of salad bars in elementary schools. The results support advocates and policy recommendations for promoting salad bars as a mechanism to increase elementary students’ FV consumption.

## Supplementary Information

Supplementary Material 1.

## Data Availability

We will accept data and materials requests from qualified investigators within the greater scientific community after consultation with the Co-Is. All requests for data will be reviewed and approved by the investigators prior to the release of data. Shared datasets will be free of identifiers or variables that would permit linkage to or lead to disclosure of the identity of individuals. All data-sharing procedures will be in compliance with institutional and IRB policy at Arizona State University.

## Abbreviations

SB: Salad Bar Only arm
Mktg: Marketing Only arm
SB + Mktg: Salad Bar and Marketing arm
NSLP: National School Lunch Program
FV: Fruit and vegetable
COVID-19: Coronavirus 2019
HHFKA: Healthy Hunger Free Kids Act
USDA: United States Department of Agriculture
