# Supplementary material for: A cluster randomized factorial trial of school-lunch salad bars and marketing on elementary students’ objectively measured fruit and vegetable consumption
Source: Int J Behav Nutr Phys Act. 2025 May 26;22:58. doi: 10.1186/s12966-025-01758-z (PMC12105326; doi:10.1186/s12966-025-01758-z)
Supplement: Supplementary file 1 — Supplementary Material 1. [file 12966_2025_1758_MOESM1_ESM.docx]

Supplementary Table A. Comparison of Demographic Characteristics of All Students at Sampled Schools to Students in Analyzed Sample

|  | All students in sampled schools  (*N* = 4839) |  | Observations in analyzed sample  (*n* = 3080) | | | |
| --- | --- | --- | --- | --- | --- | --- |
|  |  |  | Wave 1 | Wave 2 | Wave 3 | Overall |
| Characteristic | *n (%)* |  | *n (%)* | *n (%)* | *n (%)* | *n (%)* |
| Gender |  |  |  |  |  |  |
| Female | 2331 (48.4) |  | 470 (46.8) | 500 (48.9) | 524 (49.8) | 1494 (48.5) |
| Male | 2487 (51.6) |  | 535 (53.2) | 523 (51.1) | 528 (50.2) | 1586 (51.5) |
| Missing | 21 (0.4) |  | 0 (0.0) | 0 (0.0) | 0 (0.0) | 0 (0.0) |
| Grade |  |  |  |  |  |  |
| 1^st^ | 932 (19.3) |  | 159 (15.8) | 160 (15.6) | 183 (17.4) | 502 (16.3) |
| 2^nd^ | 926 (19.1) |  | 168 (16.7) | 190 (18.6) | 202 (19.2) | 560 (18.2) |
| 3^rd^ | 939 (19.4) |  | 233 (23.2) | 235 (23.0) | 218 (20.7) | 686 (22.3) |
| 4^th^ | 1039 (21.5) |  | 220 (21.9) | 226 (22.1) | 232 (22.1) | 678 (22.0) |
| 5^th^ | 1003 (20.7) |  | 225 (22.4) | 212 (20.7) | 217 (20.6) | 654 (21.2) |
| Missing | 0 (0.0) |  | 0 (0.0) | 0 (0.0) | 0 (0.0) | 0 (0.0) |
| Race/ethnicity |  |  |  |  |  |  |
| Non-Hispanic White | 1481 (30.7) |  | 253 (25.2) | 255 (24.9) | 279 (26.5) | 787 (25.6) |
| Hispanic, White | 1775 (36.8) |  | 441 (43.9) | 410 (40.1) | 429 (40.8) | 1280 (41.6) |
| Hispanic, Unknown race | 621 (12.9) |  | 120 (11.9) | 164 (16.0) | 154 (14.6) | 438 (14.2) |
| Hispanic, Non-White or > 1 race | 265 (5.5) |  | 61 (6.1) | 60 (5.9) | 58 (5.5) | 179 (5.8) |
| American Indian/Alaska Native | 142 (2.9) |  | 28 (2.8) | 40 (3.9) | 30 (2.9) | 98 (3.2) |
| Asian or Pacific Islander | 130 (2.7) |  | 27 (2.7) | 20 (2.0) | 35 (3.3) | 82 (2.7) |
| Black/African American | 406 (8.4) |  | 75 (7.5) | 74 (7.2) | 67 (6.4) | 216 (7.0) |
| Missing | 19 (0.4) |  | 0 (0.0) | 0 (0.0) | 0 (0.0) | 0 (0.0) |
| Free/Reduced-price lunch |  |  |  |  |  |  |
| Paid | 1492 (31.5) |  | 213 (21.2) | 186 (18.2) | 223 (21.2) | 622 (20.2) |
| Free/Reduced-price lunch | 3245 (68.5) |  | 792 (78.8) | 837 (81.8) | 829 (78.8) | 2458 (79.8) |
| Missing | 102 (2.1) |  | 0 (0.0) | 0 (0.0) | 0 (0.0) | 0 (0.0) |
